# Correlation between the Warburg effect and progression of triple-negative breast cancer

**DOI:** 10.3389/fonc.2022.1060495

**Published:** 2023-01-27

**Authors:** Shaojun Liu, Yuxuan Li, Meng Yuan, Qing Song, Min Liu

**Affiliations:** Department of Oncology, Suzhou TCM Hospital Affiliated to Nanjing University of Chinese Medicine, Suzhou, China

**Keywords:** Warburg effect, glycolysis, metabolic plasticity, triple-negative breast cancer, basal-like breast cancer

## Abstract

Triple-negative breast cancer (TNBC) is ineligible for hormonal therapy and Her-2-targeted therapy due to the negative expression of the estrogen receptor, progesterone receptor, and human epidermal growth factor receptor-2. Although targeted therapy and immunotherapy have been shown to attenuate the aggressiveness of TNBC partially, few patients have benefited from them. The conventional treatment for TNBC remains chemotherapy. Chemoresistance, however, impedes therapeutic progress over time, and chemotherapy toxicity increases the burden of cancer on patients. Therefore, introducing more advantageous TNBC treatment options is a necessity. Metabolic reprogramming centered on glucose metabolism is considered a hallmark of tumors. It is described as tumor cells tend to convert glucose to lactate even under normoxic conditions, a phenomenon known as the Warburg effect. Similar to Darwinian evolution, its emergence is attributed to the selective pressures formed by the hypoxic microenvironment of pre-malignant lesions. Of note, the Warburg effect does not disappear with changes in the microenvironment after the formation of malignant tumor phenotypes. Instead, it forms a constitutive expression mediated by mutations or epigenetic modifications, providing a robust selective survival advantage for primary and metastatic lesions. Expanding evidence has demonstrated that the Warburg effect mediates multiple invasive behaviors in TNBC, including proliferation, metastasis, recurrence, immune escape, and multidrug resistance. Moreover, the Warburg effect-targeted therapy has been testified to be feasible in inhibiting TNBC progression. However, not all TNBCs are sensitive to glycolysis inhibitors because TNBC cells flexibly switch their metabolic patterns to cope with different survival pressures, namely metabolic plasticity. Between the Warburg effect-targeted medicines and the actual curative effect, metabolic plasticity creates a divide that must be continuously researched and bridged.

## 1 Introduction

Global cancer statistics reported that by the end of 2021, female breast cancer incidence had surpassed that of lung cancer, ranking first in the world and being the leading cause of female cancer death (1). Breast cancer is classified into three subtypes based on the expression of estrogen receptor (ER), progesterone receptor (PR), and human epidermal growth factor receptor-2 (Her-2). The luminal A subtype (ER positive, PR positive, Her-2 negative), the luminal B subtype (ER positive, PR positive, Her-2 negative or positive), the Her-2 subtype (ER negative, PR negative, Her-2 positive), and the basal-like subtype (ER negative, PR negative, Her-2 negative) (2, 3). The basal-like subtype overlaps with triple-negative breast cancer (TNBC) (4) and is the most aggressive subtype of breast cancer, with a higher recurrence rate, tremendous metastatic potential, and shorter overall survival, and is generally associated with a higher cancer burden and poorer disease outcomes (5–7). The lack of relevant receptor expression means that TNBC has lost its candidate status for both hormone therapy and Her-2-targeted therapy (8, 9). Despite significant advances in developing emerging targets in recent years, few patients have benefited from them, and chemotherapy remains the current standard of care (10–12). However, evolving chemoresistance has hampered treatment progress, and chemotherapy toxicity has significantly increased the patient burden (13, 14). The Warburg effect may be a promising therapeutic entry point for the immediate introduction of more effective treatment procedures for the clinical management of TNBC.

As is common knowledge, cancer cells carry out metabolic reprogramming to deal with the survival pressures derived from several aspects, such as the immune system, chemotherapeutics, and anoikis. Glucose metabolism rewiring is a significant part of this process, manifested as tumor cells maintaining a high-flux glycolysis phenotype rather than oxidative phosphorylation (OXPHOS) even under normoxia conditions, known as the Warburg effect (or aerobic glycolysis) (15, 16). The Warburg effect was postulated by the team of Otto Warburg, who found that tumor cells allocate 66% of glucose under oxygen availability conditions for fermentation and the remainder for respiration (17). Impaired mitochondrial structure and function in tumor cells is the initial explanation for this phenomenon (18). A recent study demonstrated a high frequency of mitochondrial defects in TNBC cells, resulting in a metabolic bias towards glycolysis and the promotion of cancer metastasis (19). On the contrary, Reda A. et al. suggest that the mitochondria of tumor cells function normally and serve as a protective mechanism against glycolysis inhibition (20). Current opinion prefers the latter, where the metabolic phenotypes of tumor cells are plastic. However, its regulatory agencies have not been entirely clarified (21).

The emergence of the Warburg phenotype is in line with an evolutionary model. The localized hypoxic microenvironment in which the pre-malignant lesion is located creates a selective pressure that drives its cellular metabolism to glycolysis. Mutations and epigenetic modifications allow glycolysis to break through hypoxic constraints, leading to its constitutive expression (22, 23). Furthermore, hypoxia-induced remodeling of the TNBC extracellular matrix (ECM) positively affects the development of the Warburg phenotype (24). Evidence suggests that the Warburg effect participates in TNBC invasive behaviors, including proliferation, metastasis, recurrence, drug resistance, and immune escape (25–28). The up-regulation of TNBC aerobic glycolysis is a high-probability event in breast cancer subtypes (29, 30). The enhanced reliance of TNBC stem cells on the Warburg effect supports their aggressive phenotype (31). However, there are always two sides to the coin. While the Warburg effect confers TNBC with a higher degree of malignancy, it simultaneously creates opportunities for metabolic-targeted therapeutic strategies (32). In addition, the Warburg effect flux and its product abundance have been used to evaluate the prognosis of TNBC patients receiving glycolysis inhibitors (33). Interestingly, not all TNBC cells are sensitive to glycolysis inhibitors (34), indicating that different states of TNBC cells differ from their choice of glycolytic pathways (35), which is consistent with the metabolic plasticity theory. This theory is strongly supported by the fact that mitochondrial copper depletion drives the MDA-MB-231 cells to reorient their metabolism from mitochondrial respiration to glycolysis to suppress TNBC translocation (36, 37). Consequently, precision medicine requires the customized and stratified management of TNBC patients based on their metabolic directivity.

## 2 Hypoxia is an incubator for the Warburg phenotype in TNBC

### 2.1 Hypoxia breeds the Warburg phenotype *via* forming selective pressure

**Figure 1:**
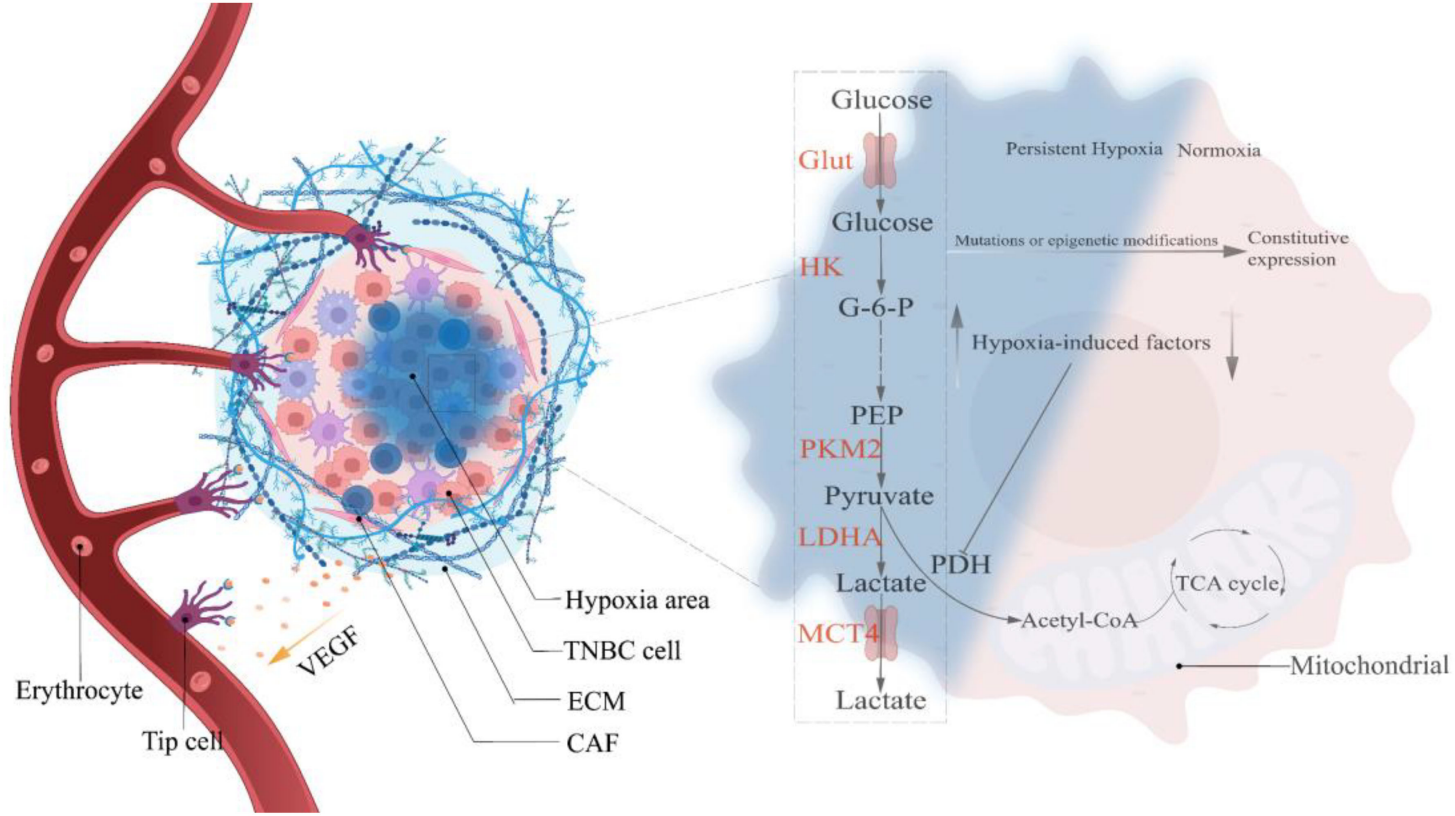
Hypoxia selects the Warburg phenotype. Hypoxia-mediated angiogenesis is gradual in precancerous lesions, and the creation of persistent hypoxia induces the Warburg phenotype to become the predominant metabolic pathway in TNBC cells. Continuous mutations and epigenetic modifications throughout the growth of cancers lead glycolysis to exceed the oxygen concentration restriction, resulting in the constitutive expression of the Warburg phenotype. HIF, Hypoxia-induced factor; VEGF, Vascular endothelial growth factor; Glut, Glucose transporter; HK, Hexokinase; PKM2, pyruvate kinase isozyme typeM2; LDHA, Lactate dehydrogenase; MCT4, Monocarboxylate transporter 4; G-6-P, Glucose-6-phosphate; PEP, Phosphoenolpyruvate; Acetyl-CoA, Acetyl-coenzyme A.

Hypoxia-mediated Warburg-like metabolic changes are analogous to the Darwinian evolutionary model (23). Epithelial cells acquiring oncogenic transformation is the first step in breast carcinogenesis, whether under the stochastic or stem cell hypothesis (38). Upon reciprocal touch, non-oncogenic breast epithelial cells abide by contact inhibition and halt growing and reproducing (39). However, cells with oncogenic transformation evade contact inhibition (40), continuing to grow and proliferate in a compacted epithelial environment until apical extrusion occurs, completing the transition from monolayer to multilayer epithelium under the coordination of the CD44/COL17A1 pathway and progressively developing into pre-malignant lesions (41) which are separated from the surrounding matrix by a nearly continuous layer of myoepithelial and basement membrane proteins (42). Therefore, oxygen in the vascular matrix must traverse the basement membrane and tumor cell layer. Studies have indicated a limiting distance for oxygen diffusion (approximately 150µm), pre-malignant lesions with uncontrolled proliferation are bound to exceed this diffusion limit, resulting in a hypoxic region (23). Under normoxic conditions, hypoxia-inducible factors (HIFs) are rapidly degraded by prolyl hydroxylation of EglN family members, whereas hypoxia inhibits EglN1 activity, resulting in a massive accumulation of HIFs. In addition, glutamate secretion from TNBC causes EglN1 to undergo oxidative self-inactivation, which can also prevent HIFs from being degraded (43). Studies have shown that HIF directly or indirectly up-regulates the expression of critical glycolysis regulatory elements, resulting in metabolic adaptive transformation (OXPHOS to glycolysis), and it simultaneously stimulates angiogenesis to prepare for eventual vascular normalization. However, angiogenesis invariably lags behind, exposing tumor cells to persistent hypoxic stress (44, 45). Only glycolytic tumor cells, according to the evolutionary models, can survive in a constant hypoxic environment. Mutations or epigenetic modifications during selection lead to a glycolytic constitutive expression (manifested as the removal of oxygen concentration limitation) that is up-regulated in response to specific survival pressures, thereby conferring a survival advantage to tumor cells **(** **Figure 1** **)** (23). The “specific survival pressures” argument is based on the metabolic plasticity doctrine, which holds that the dominant metabolism maintained by tumor cells must be elastic in response to variations in survival pressures (15). For example, TNBC, which keeps a hybrid metabolic phenotype, activates the AMPK pathway to enhance OXPHOS when exposed to glycolytic inhibitor toxicity and vice versa (21). In addition, the 3D model of TNBC developed by Liu C. et al. revealed that the tumor tissue exhibits escalating matrix stiffness from the core to the periphery, and the dominant metabolism also manifests a distribution from the core glycolysis to the peripheral OXPHOS and fatty acid metabolism (46). Microenvironmental acidification and glucose limitation as consequences of up-regulation of glycolysis will further select tumor cells with survival advantages (47–50).

### 2.2 Hypoxia breeds the Warburg phenotype *via* inducing ECM remodeling

**Figure 2:**
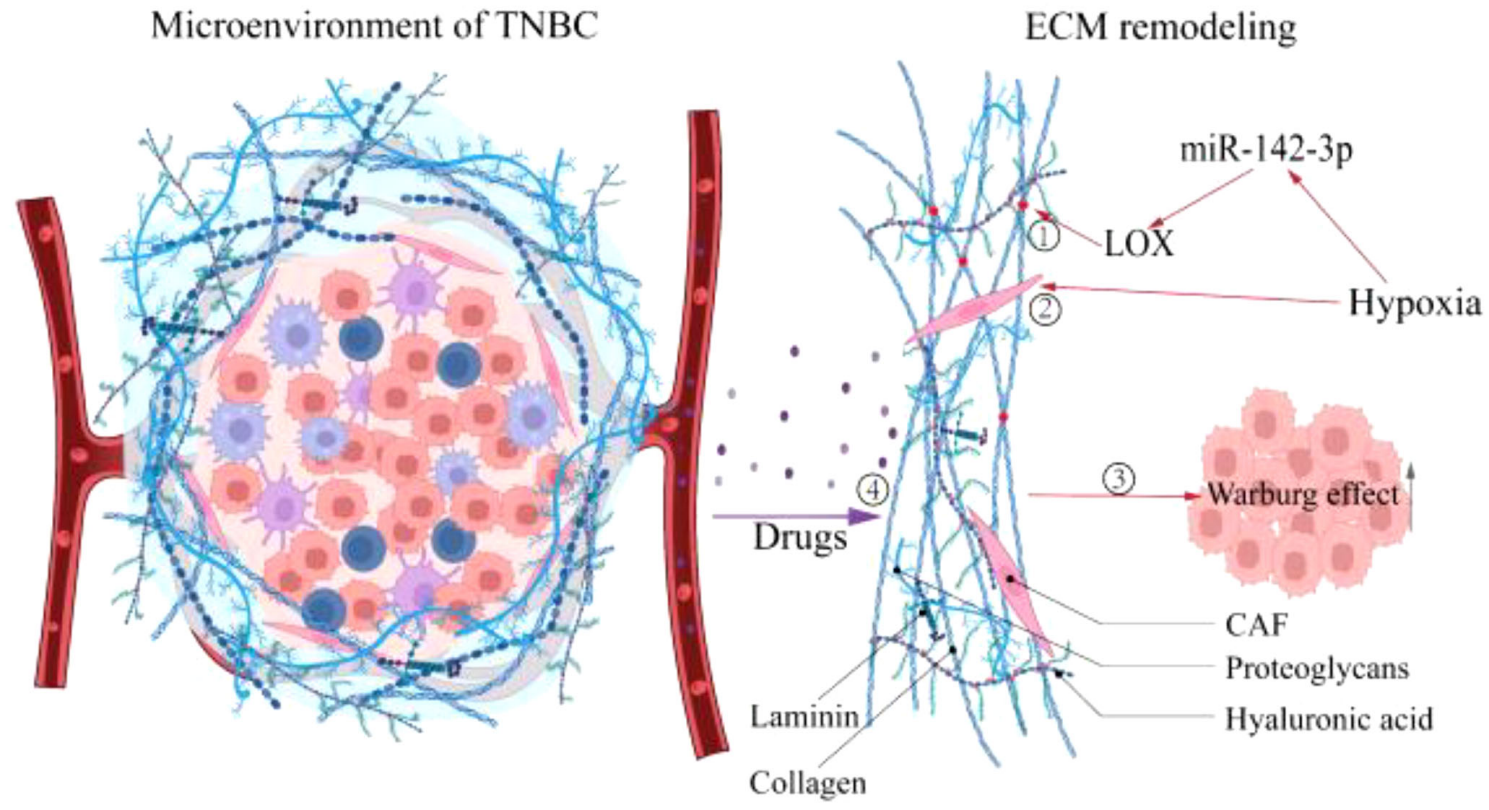
Hypoxia promotes the ECM remodeling. Hypoxia causes ECM remodeling, which affects tumor progression further. ①Enhanced matrix cross-linking. ②Local compaction and distant stretching of matrix fibers derived from CAFs-mediated contractile forces. ③ECM remodeling promotes the Warburg effect. ④ECM stiffness prevents drug penetration. LOX, Lysyl oxidase; CAF, Cancer-associated fibroblast.

ECM, a substantial 3D supramolecular entity composed of hundreds of different building blocks, is an essential component of TNBC, and its changes profoundly affect the fate of TNBC cells (51, 52). Divergent matrix-remodeling strategies mediated by Mmp14/MT1-MMP define the branching morphology of developing and neoplastic epithelial cells early in breast carcinogenesis (53). This process may involve the effect of matrix remodeling on the chromatin state of mammary epithelial cells (54). On the other hand, ECM stimulates tumor growth in various ways throughout the development of TNBC. For instance, ECM compliance controls the cellular response to contact guidance by generating population transfer between round and elongated cells, hence affecting the migratory dynamics of tumor cells (55). Consequently, ECM remodeling plays a crucial role in carcinogenesis and progression. The preceding section identified hypoxia as an early event in TNBC. Interestingly, current research indicates hypoxia as a potential trigger for ECM remodeling (56, 57). Increased matrix proteins due to disruption of ECM deposition and degradation homeostasis (58), enhanced matrix cross-linking driven by lysyl oxidase (LOX) (59), and local compaction and distant stretching of matrix fibers derived from CAFs-mediated contractile forces (60, 61) are direct behaviors that cause ECM remodeling. Studies have demonstrated that hypoxia affects the aforementioned behaviors in a HIF-dependent manner (57), such as HIF-1α/miR-142-3p/LOX pathway (62) and the HIF-1α/CAF pathway (63), ultimately leading to changes in ECM biophysical and biological properties. Notably, a recent study in which ECM remodeling activated the glycolytic pathway in murine 4T1 cells suggests that ECM is involved in regulating the metabolism of TBNC cells (64). Concerning its regulation mechanism, Sullivan WJ et al. determined that ECM remodeling boosted lactate generation in TNBC cells *via* the Zfp36/TXNIP/Glut1 signaling pathway (65). Additionally, connective tissue growth factor (CTGF) in conjunction with ECM deposition accelerated TNBC glycolysis *via* integrin β3/FAK/Src/NF-B P65/Glut3 signaling (66). Moreover, ROCK isoforms differentially regulate the RhoA/ROCK1/p-MLC and RhoA/ROCK2/p-cofilin pathways through integrin β1-activated FAK signaling in an ECM stiffness-dependent manner to regulate MDA-MB-231 cell motility (67), and Rho/ROCK signaling can stimulate Glut1 translocation to the PM and enhance extra-membrane glucose uptake TNBC cells (68). In summary, hypoxia promotes matrix remodeling in various ways that further influence the metabolic pattern of TNBC cells **(** **Figure 2** **)**, which simultaneously provides suggestions for developing innovative ECM-targeting treatment approaches (69).

## 3 The Warburg effect contributes to the progress of TNBC

### 3.1 The Warburg effect with TNBC proliferation

Growth factor stimulation of TNBC cells commences the proliferation program (70), and cell proliferation undergoes three periods: I. interphase; II. mitosis; and III. cytokinesis which is called the cell cycle. The interphase accounts for about 90% of the time required for the entire cell cycle and is divided into three phases, namely G1, S, and G2. The cell cycle is precisely modulated by checkpoints such as cyclin-cyclin-dependent kinase complex (cyclin-CDK) and CDK inhibitors (examples include P15, P21, P27), inactivation or mutation of anti-oncogenes, and overexpression or amplification of proto-oncogenes leading to cell cycle checkpoint disorders and ultimately to uncontrolled cancer cell proliferation (71, 72). Positive cell cycle regulation of the Warburg effect is generally achieved through cell cycle checkpoints (73), such as critical glycolytic kinase 6-phosphoglucose-2-kinase (PFKFB3), pyruvate kinase (PKM2) was shown to promote cell cycle (72). Mechanistically, PFKFB3 is transported to the nucleus, and its products F2 and 6BP activate CDKs. Moreover, F2 and 6BP induce degradation of the CDK-mediated G1/S transition inhibitor P27 (74, 75). PKM2 binds to β-linked proteins to form the PKM2-β-linked protein complex to up-regulate cell cycle protein D1 to drive the G1/S transition (72). A study targeting PKM2 to regulate cell cycle progression and inhibit TNBC invasion suggests that further study of the interaction between the Warburg effect and cell cycle is a concern in TNBC management (76).

### 3.2 The Warburg effect with TNBC metastasis

Tumor metastasis starts from the early stage of the tumor and continues until the cancer is removed. It can be divided into three periods: dissemination, metastatic dormancy, and host organ colonization (77). This process is not a simple linear cascade; concurrency and overlap are more prevalent (78). During dissemination, metastasis-initiating cells (MICs), which are assumed to be derived from cancer stem cells (CSCs) or CSC-like cells, lose their epithelial features and acquire mesenchymal traits through epithelial-mesenchymal transition (EMT) (79, 80), primarily manifested by downregulation of E-calmodulin and reduced cell adhesion, in which the Warburg effect plays an instrumental role (81). For example, high expression of Y-box binding protein 1 (YBX1) was detected in TNBC, which up-regulates aerobic glycolytic flux to accelerate EMT progression (82). Immediately afterward, MICs with mesenchymal properties detach from the tumor bed as single cells or cell clusters and become circulating tumor cells (CTCs) (79). In fact, CTC clusters are more conducive to tumor metastasis (83), so primary tumors significantly up-regulate HIF-1α-mediated Desmoglein2 (DSG2) to promote the formation of CTC clusters (84).

**Figure 3:**
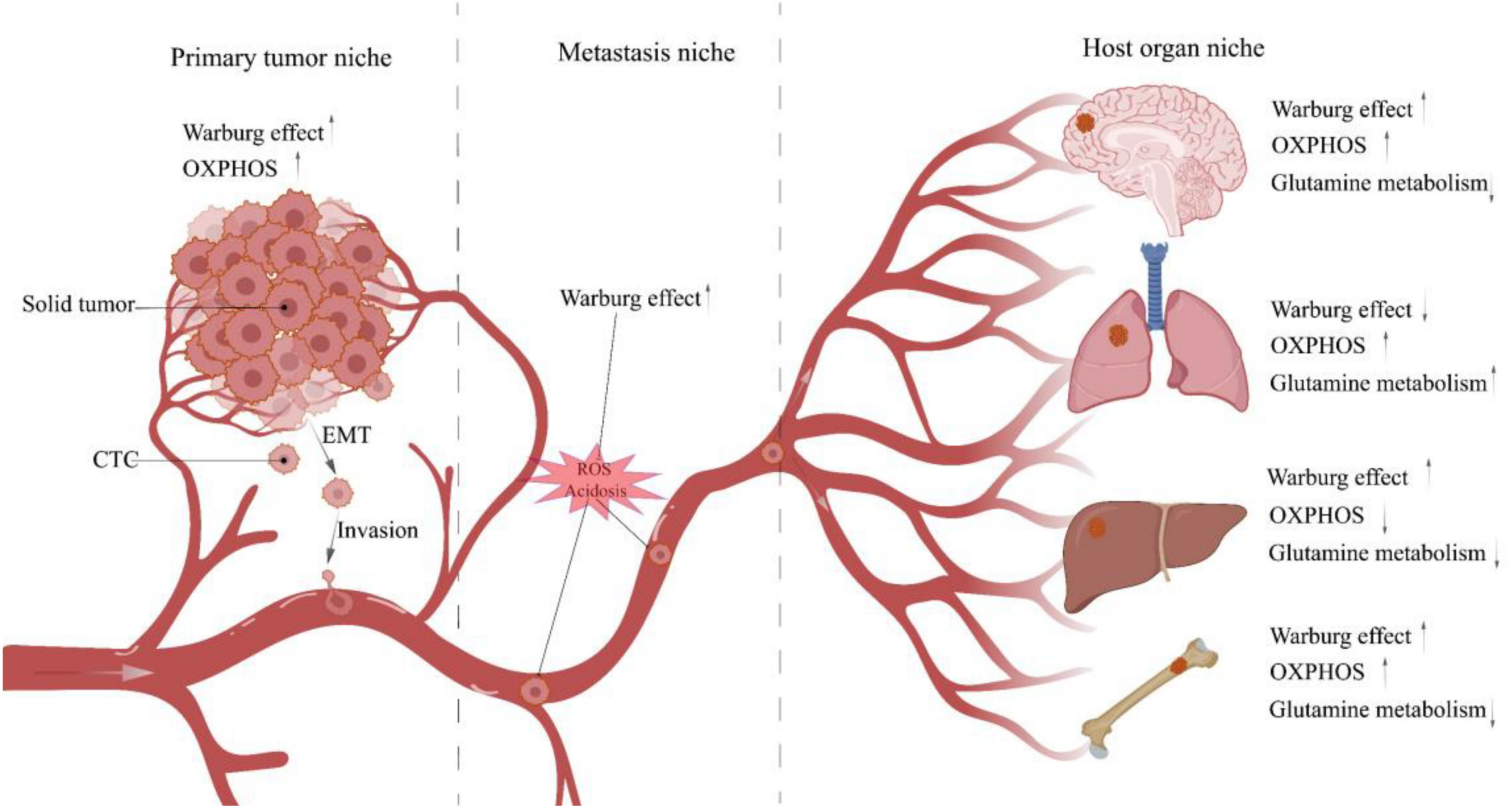
Metabolic characteristics of TNBC cells in different niches. When tumor cells leave the tumor bed and begin the metastasis process, they will traverse multiple niches with diverse microenvironments. Different microenvironments impose distinct survival restrictions, and tumor cells must change their metabolic pathways dynamically to resist these pressures. CTC, Circulating tumor cell; EMT, Epithelial-mesenchymal transition.

Subsequently, CTCs enter the TME, and the acidic TME shaped by the accumulation of glycolytic acid metabolites intensifies CTCs migration (85, 86). In addition, Glut3 overexpression in TNBC induces activation of M1 tumor-associated macrophages (M1-TAM) *via* lactate/C-X-C motif Chemokine ligand 8 (CXCL8), consequently creating an inflammatory TME that promotes CTCs metastasis (87). Notably, TNBC delivers Integrin beta 4 (ITGB4) to CAFs located in TME through exosomes and initiates the glycolytic phenotype of CAFs *via* BNIP3L-dependent mitophagy (known as the reverse Warburg effect) (88). Its product lactate shuttles between CAFs and CTCs *via* MCT4/MCT1 as a metabolic coupling link and activates the TGFβ1/P38 MAPK/MMP2/9 signaling axis to up-regulate CTCs’ mitochondrial activity and promote metastasis (89).Even more to the point, glycolysis is not the sole up-regulated metabolic phenotype in cancer cell migration. Other metabolic patterns, such as OXPHOS, fatty acid metabolism, and glutamine metabolism, are similarly up-regulated under certain conditions (90). And the metabolic phenotype of CTCs is dynamically remodeled at different stages of tumor metastasis to help them manage the changing microenvironment of the metastatic cascade and ensure a successful transition **(** **Figure 3** **)** (91). Next, CTCs migrate towards the vasculature, and the Glut1/integrin β1/Src/focal adhesion kinase (FAK) signaling pathway activated by PCB29-P exposure may be involved in this process (92). CTCs entering the blood circulation are challenged by ROS, and high concentrations of ROS are cytotoxic. At this time, the advantages of CTCs clusters gradually emerge, which induce microenvironmental hypoxia and activate HIF-1α-mediated glycolysis in response to oxidative stress (93). Moreover, when CTC reaches the bloodstream and is threatened by Anoikis, only a few Anoikis-resistant tumor cells survive (94). And the phosphorylation and activation of lactate dehydrogenase (LDHA), a glycolytic critical kinase, has been discovered to boost Anoikis resistance (95).

Next is an overlap of host organ colonization and metastatic dormancy, in which the majority of tumors metastasize to specific organs, called “organotropism” (96). Studies have shown that the lung, bones, and brain are more likely to serve as TNBC target organs (97, 98). After reaching the targeted organ, CTCs occupy the favorable niche and become disseminated tumor cells (DTCs). Due to the balance between tumor cell proliferation and apoptosis, DTCs enter the reversible growth arrest of single cells located in the G0/G1 phase of the cell cycle, called cell dormancy. This persists for years or even decades, and DTCs reactivate when the balance swings toward cell proliferation, either because of proangiogenic substances or because tumor cells defy immune suppression (77, 79, 99). For instance, CD39 PD-1 CD8 T cells were demonstrated to mediate metastatic dormancy in TNBC lung-oriented DTCs, and depletion of CD8 T cells may induce DTCs reactivation (100). Activated DTCs destroy host organs and form metastases, such as up-regulation of the Warburg effect by MDA-MB-231 cells found in bone TME to produce excessive lactate, leading to MCT4/MCT1-mediated osteolytic lesions (101).

### 3.3 The Warburg effect with TNBC recurrence

Minimal residual disease (MRD) is considered the basis of TNBC recurrence (102), and postoperative circulating tumor DNA (ctDNA) sequencing and CTCs counting have been used to detect MRD and evaluate the likelihood of patients suffering from recurrence (103). Cell dormancy is critical for MRD survival and reactivation (104, 105). Genetic heterogeneity, TME regulation, ecological niche, and immunosuppression drive MRD into a dormancy (106) characterized by cell cycle arrest, ecological niche dependence, drug resistance, immune escape, and reversibility (107). When conditions such as surgery, drugs, and metabolic remodeling intervene, MRD exits dormancy and causes recurrence (108). Glycolysis is possibly involved in the reactivation of dormant MRD. Studies have shown that dormant cells exhibit an increase in mitochondrial mass and mitochondrial ROS. Therefore, dormant cells inhibit oxidative stress-induced apoptosis *via* autophagy-related 7 (ATG7) mediated autophagy (109), in which up-regulated AGT7 suppresses the glycolytic phenotype (110). During the transitional period of dormant cells toward reactivation, autophagic inhibition and increased abundance of PFKFB3, a key mediator of glycolysis, facilitate dormant escape (111). The argument that a negative correlation between autophagy and the Warburg phenotype differs from Radic Shechter et al., who found that MRD retains the metabolic memory of original tumor cells and maintains high aerobic glycolytic flux to ensure survival (112). A possible explanation is that MRDs dynamically reshape the balance between autophagy and glycolysis in response to varied ecological niches (113). In addition, consistent with the primary tumor, the metabolism of the cells that undergo macroscopical metastasis after escaping the dormant state continues to be biased toward glycolysis to sustain growth (114). Remarkably, the tricarboxylic acid cycle (TCA cycle) metabolites succinate, fumarate, and 2-hydroxyglutarate (2-HG) were associated with post-treatment tumor reconstruction (115), and glycolytic product lactate functions as a substrate to support TCA cycle running (116).

As evident from recent reviews (106, 108, 117), despite the emerging roles of the Warburg phenotype in metastatic dormancy of tumor cells, there is a considerable blankness in the relevant research, including how the Warburg phenotype controls DTCs to enter a dormancy state? How does the Warburg phenotype help dormant DTCs survive? Furthermore, how the Warburg phenotype regulates dormant DTCs reactivation? Metastatic dormancy is essential for DTCs survival and reactivation as a protective mechanism (118), so filling these gaps may provide new clinical opportunities for TNBC.

### 3.4 The Warburg effect with TNBC immune escape

**Figure 4:**
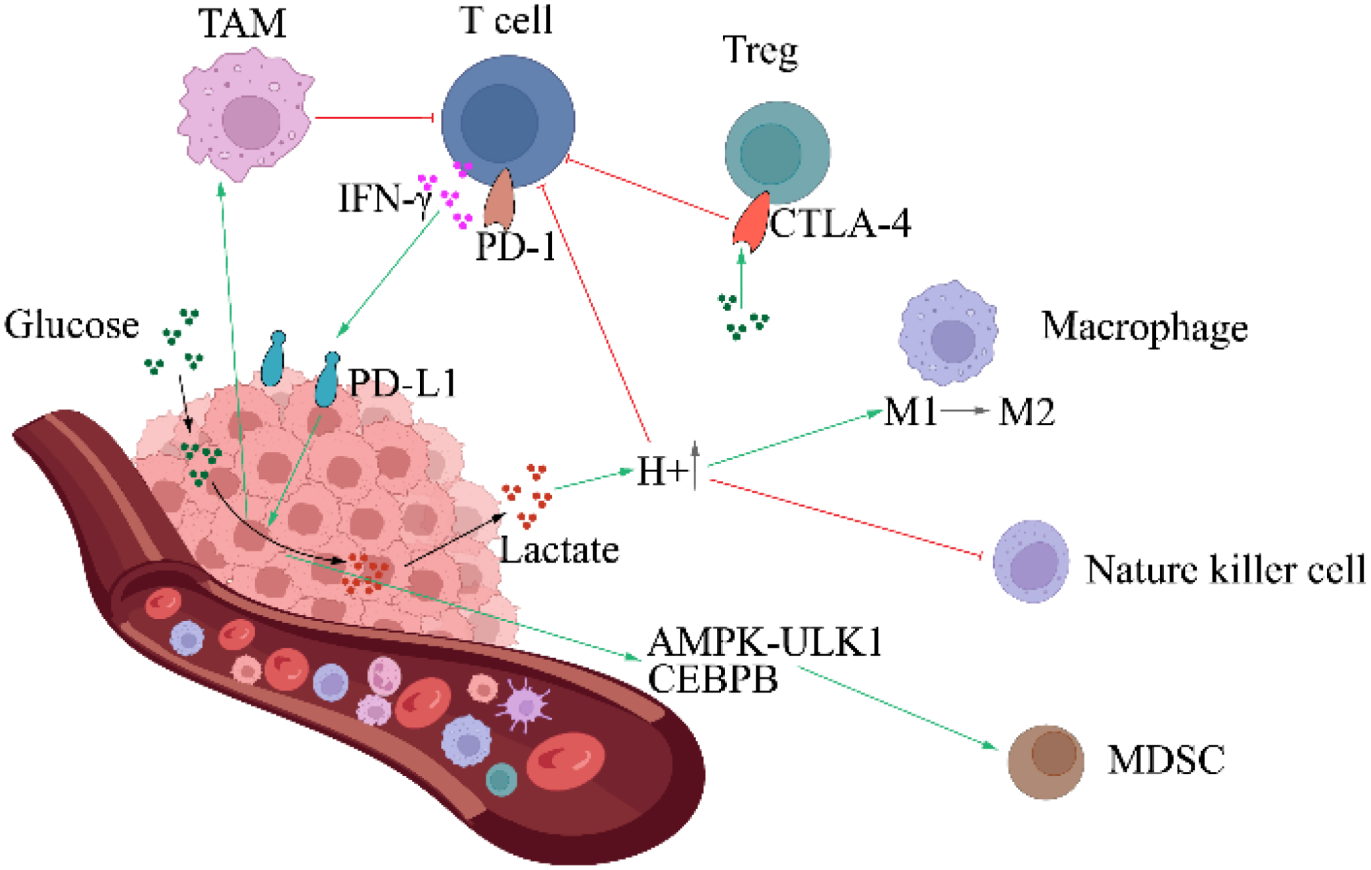
The Warburg effect enhances immune escape of TNBC. Glycolysis facilitates tumor immune evasion *via* multiple mechanisms. MDSC, Myeloid-derived suppressor cell.

From the above discussion, it can be clarified that while TNBCs adaptively modulate their metabolic phenotype following different conditions, TNBCs up-regulate the Warburg phenotype to maintain their invasion activity is still a high probability event. Accordingly, high glycolytic flux means an adequate supply of extra-membrane glucose is necessary. Increasing evidence that most immune cells present in TME **(** **Figure 4** **)**, including innate immune cells such as NK cells, macrophages, and dendritic cells; adaptive immune cells, such as T cells in the activated state, mimic the metabolic patterns of tumor cells (119, 120). Therefore, the competition between tumor cells and immune cells for metabolic substrates is inevitable (121), and the consequence is always that immune cell function is curbed due to the lack of fuel (122). Because tumor cells will induce immune cells’ glycolytic suppression, for example, IFN-γ secreted by T cells or the glycolytic phenotype of the tumor itself triggers PD-L1 expression in tumor cells. In turn, PD-L1 mediates up-regulation of the glycolytic pathway in tumor cells *via* the Akt/mTOR pathway, imposing a nutrient limitation on T cells. Prolonged exposure to glucose limitation will lead to irreversible functional impairment that cannot be corrected by simple nutrient supplementation (122, 123). In addition, the expression of CTL-4 associated with immune suppression and its principal ligand CD80/CD86 were detected in TNBCs (124), and a study of CTL-4 blockade by ipilimumab linked Treg instability to its glycolysis, suggesting the involvement of CTL-4 in metabolic competition between TNBCs and immune cells (125).

Lactate mediates multiple pro-tumor behaviors, including immune escape (126, 127). For instance, lactate works directly with mitochondrial antiviral signaling proteins (MAVS), attenuates retinoic acid-inducible gene I-like receptors (RLRs) signal transduction and downstream type I interferons (IFNs) production, and induces failure of immune surveillance (128). Unfortunately, this mechanism has not been characterized in TNBC cell lines, but it is a potential research direction. Furthermore, the acidic microenvironment due to lactate accumulation significantly down-regulates macrophage-mediated Programmed Cell Removal (PrCR) damage to MDA-MB-231 cells (129).

Indeed, the Warburg phenotype derived from TNBC can also evade immune surveillance by activating immunosuppressive cells. For example, tumor glycolysis regulates the molecular network of AMPK-ULK1, autophagy, and CEBPB pathways. This way, myeloid-derived suppressor cells (MDSC) are activated, and immune suppression is maintained (130). Moreover, Microenvironmental lactic acidosis induces tumor-associated macrophages (TAM) to skew toward an M2-like phenotype, resulting in the obstacle of immuno-detection of tumor cells by macrophages (129, 131). A recent study found that TNBC CTCs recruit neutrophils to form CTC-neutrophil clusters, leading to an accelerated metastatic process (132). However, further studies are needed to determine whether the Warburg phenotype is involved in neutrophil recruitment and whether CTC-neutrophil clusters contribute to immune evasion (133).

### 3.5 The Warburg effect with TNBC multi-drug resistance

Currently, chemotherapy based on anthracyclines and taxanes is the standard treatment for TNBC. The effectiveness of platinum compounds in neoadjuvant chemotherapy has been confirmed by evidence. Research on drugs related to targeted therapy, immunotherapy, and endocrine therapy has also collected initial results (134, 135). Nonetheless, multi-drug resistance deepens the conflict between TNBC therapy options and adverse outcomes (136). Studies have indicated that primary resistance genes pre-exist and are adaptively selected by treatment regimens. Acquired resistance gene transcriptional profiles are obtained by reprogramming in response to treatment in TNBC patients (137). Various mechanisms mediate drug resistance in TNBC (138), including the Warburg phenotype (25, 139, 140). First, the Warburg effect confers enhanced DNA repair capacity to tumor cells. Rac1 enhances nucleotide production to defend MDA-MB-231 cells from chemotherapeutic drug-induced DNA damage by triggering the aldolase A and ERK signaling cascades and upregulating aerobic glycolysis, especially its branching Pentose phosphate pathway (PPP) fluxes (141). A recent study demonstrating that overactivation of the ERK pathway promotes apoptosis in TNBC cells shows the dual role of ERK in TNBC biology, which should therefore be considered when selecting ERK as a therapeutic target (142). Second, the Warburg effect gives tumor cells a more robust antioxidant capacity. ROS up-regulation induced by chemotherapy drugs exposed tumor cells to oxidative stress, oxidative damage provoked the overexpression of the PPP rate-limiting enzyme glucose-6-phosphate dehydrogenase (G6PD), accelerated PPP enhanced the production of nicotinamide adenine dinucleotide phosphate (NADPH), thereby up-regulating the synthesis rate of the antioxidant glutathione (GSH). Ultimately, the oxidative damage caused by ROS can be inhibited (143). In addition, autophagy’s clearance of injured organelles and macromolecules is essential for inhibiting drug-induced apoptosis in TNBCs (144, 145). A recent study identified Proviral Insertion in Murine Lymphomas 2 (PIM2) directly binding to hexokinase-II (HK-2) and phosphorylating HK2 on Thr473. Phosphorylated HK2 up-regulates glycolytic flux and induces autophagy to exert chemoresistance by promoting protein stability (146). The Warburg effect also manifests resistance by inhibiting drug influx and promoting drug efflux. Glycolysis generates a large amount of H^+^ accumulation in the cytoplasm, and if it is not removed in time, the consequent acidosis threatens cell survival. The existence of a net acid extrusion system ignores such events. It leads to the rise of intracellular pH (pHi) and the reduction of extracellular pH (pHe) simultaneously. This pH gradient has been confirmed to be conducive to the formation of drug resistance (147). Elevated pHi not only inhibits the influx of alkaline drugs such as doxorubicin but also induces active drug efflux by coordinating the transporter ABCB1 (148). In addition, ABC transporters are ATP-dependent (149), and suppressing glycolysis leads to transporter inactivation (150), so it is reasonable to speculate that glycolysis provides energy support for the actions of ABC transporters. Based on the above theory, Omran Z et al. recommend that the intervention of proton pump and transporter inhibitors is vital to manage resistance (151).

Notably, with the intervention of compounds other than chemotherapy, the resistance spectrum of TNBC expands, such as lactate modulating anti-PD-1/PD-L1 drug activity (152) *via* activating TAMs (153). In summary, targeting the Warburg effect seems perfect for curbing resistance; however, this is not the case; silencing of GLUT1 does not ablate TNBC resistance as expected but further induces drug resistance *via* Akt/GSK-3β/β-catenin/survivin signaling (154). This opposite phenomenon may involve dynamic remodeling of metabolism (155) since switching metabolism to mitochondrial or lipid metabolism equally mediates resistance, depending on the molecular phenotype of the interventional drug (156, 157). Hence, careful considerations should be taken into account in sequential drug resistance control programs.

## 4 Precision medicine for Warburg effect-dependent TNBC

### 4.1 Stratified management of TNBC patients based on metabolomics

In the early stages of TNBC, hypoxia gives birth to the Warburg phenotype, and mutations or epigenetic modifications result in constitutive expression of the Warburg effect. However, the pressure on TNBC’s survival is ever-changing, necessitating that it maintain a varied metabolic pattern (15). Therefore, glycolysis inhibitors are only effective against a subset of TNBCs (34). Hence, it is necessary to identify Warburg-dependent TNBC and administer tailored treatment for it. With the advent of omics approaches, several teams have stratified TNBC based on various omics analyses and obtained experimental findings (34, 158–161) **(** **Table 1** **)**. However, the road is always tortuous; stratification schemes relying on genomics and transcriptomics failed to break through the barrier of LAR and BLIS tumors in later FUTURE experiments (162). Of note, Xiao Y et al. integrated metabolomics and genomics to classify TNBC into three subtypes: C1, with ceramide and fatty acid enrichment; C2, with overexpression of oxidative processes and glycosyl transfer-related metabolites; and C3, with low-level metabolic dysregulation, and proposed that genomics-based LAR subtypes overlap with C1 subtypes and BLIS subtypes overlap with C2 and C3 subtypes, finding a new solution for refractory TNBC (158, 163). Moreover, by investigating metabolic dysregulation in TNBC, Gong Y et al. identified the lipogenic subtype MPS1 with up-regulated lipid metabolism, the glycolytic subtype MPS2 with up-regulated carbohydrate and nucleotide metabolism, and the mixed subtype MPS3 with partially dysregulated pathways, each of MPS showed consistent sensitivity to inhibitors targeting the same metabolic pathway (34). Accordingly, stratified management of TNBC patients is crucial for minimizing inefficient medical treatment and reducing the cancer burden among patients (7).

**Table 1:** TNBC stratification schemes based on different omics.

Omics	Stratifications	Characteristics
**Genomics**	mesenchymal stem–like	• Sharing enrichment of genes for similar biological processes with the M subtype; Unique to the MSL are genes representing components and processes linked to growth factor signaling pathways • Targeted therapy: Dasatinib, NVP-BEZ235
	immunomodulatory	• Is enriched for gene ontologies in immune cell processes • Targeted therapy: Unknow
	basal-like 1	• The top gene ontologies are heavily enriched in cell cycle and cell division components and pathways • Targeted therapy: Veliparib, Olaparib, Cisplatin
	basal-like 2	• Displaying unique gene ontologies involving growth factor signaling • Targeted therapy: Veliparib, Olaparib, Cisplatin
	mesenchymal	• Displaying a variety of unique gene ontologies that are heavily enriched in components and pathways involved in cell motility, ECM receptor interaction, and cell differentiation pathways • Targeted therapy: Dasatinib, NVP-BEZ235
	luminal androgen receptor	• Gene ontologies are heavily enriched in hormonally regulated pathways including steroid synthesis, porphyrin metabolism, and androgen/estrogen metabolism • Targeted therapy: Bicalutamide, 17-DMAG
**Immunogenomics**	Immunity High	• Greater immune cell infiltration and anti-tumor immune activities, as well as better survival prognosis • Increased activation of apoptosis, calcium signaling, MAPK signaling, PI3K–Akt signaling, and RAS signaling
	Immunity Medium	• Between Immunity High and Immunity Low
	Immunity Low	• Depressed immune signatures • Increased activation of cell cycle, Hippo signaling, DNA replication, mismatch repair, cell adhesion molecule binding, spliceosome, adherens junction function, pyrimidine metabolism, glycosylphosphatidylinositol (GPI)-anchor biosynthesis, and RNA polymerase pathways
**Genomics & Transcriptomics**	immunomodulatory	• With high immune cell signaling and cytokine signaling gene expression • Targeted therapy: Immune checkpoint inhibitors
	luminal androgen receptor	• Characterized by androgen receptor signaling • Targeted therapy: Anti-androgen therapy, targeting ERBB2, CDK4/6 inhibitors
	basal-like and immune-suppressed	• Characterized by upregulation of cell cycle, activation of DNA repair, and downregulation of immune response genes • Targeted therapy: Platinum drugs, PARPi
	mesenchymal-like	• Enriched in mammary stem cell pathways • Targeted therapy: STAT3 inhibitors
**Metabolomics**	MPS1	• Upregulation of lipogenesis genes and lipids • Targeted therapy: lipid synthesis inhibitors
	MPS2	• Partial metabolic pathway dysregulation • Targeted therapy: Need to be explored
	MPS3	• Upregulation of glycolytic and nucleotide genes and intermediate metabolites • Targeted therapy: Glycolysis inhibitors; Combination of LDH inhibitors and ICIs
**Metabolomics & Genomics& transcriptomics**	C1	• Was featured with sphingolipids and FAs enrichments • Alternatives for refractory TNBC subtypes(LAR/BLIS)
	C2	• Was characterized by upregulated carbohydrate metabolism and oxidation reaction
	C3	• Showed mild metabolic differences compared with normal tissue

### 4.2 Targeted therapy for Warburg effect-dependent TNBC

Over the past few decades, significant progress has been made in studying compounds targeting glycolysis **(** **Table 2** **)**. Certain compounds target upstream regulators of glycolysis. For example, Cardamonin and Honokiol restricted glycolysis in MDA-MB-231 cells through the HIF-1α pathway (174, 175). Betulinic Acid inhibits glycolytic flux *via* GRP78/β-catenin/c-Myc or CAV-1/NF-κB/c-Myc signaling pathways (176, 177). Furthermore, some compounds target the critical regulators of the Warburg phenotype. Examples include GNE-140 and FX-11, which down-regulate glycolysis by blocking lactate dehydrogenase (LDH) (34). Elemene (β-elemene) suppresses breast cancer metastasis by obstructing pyruvate kinase M2 (PKM2) dimerization and nuclear translocation.

**Table 2:** Compounds targeting the Warburg effect in TNBC.

Compounds	Signaling pathways	Therapeutic strategies	References
**Hemin& metformin**	BACH1	Suppressing glycolysis and OXPHOS	(164)
**FX-11& anti-PD-1**	LDH, PD-1	Suppressing the Warburg effect and immunosuppression	(34)
**Silibinin**	EGFR-MYC-TXNIP	Inhibiting glycolysis to retard biosynthetic demands	(165)
**Marizomib& STF-31**	PGC-1α, GLUT1	Suppressing OXPHOS and Warburg effect	(166)
**Chidamide**	miR-33a-5p-LDHA	Inhibiting glycolysis to retard biosynthetic demands	(167)
**Kudingcha**	ROS	Suppressing the Warburg effect and glutamine metabolism	(168)
**Everolimus**	PI3K/AKT/mTOR	Suppressing the Warburg effect and glutamine metabolism	(169)
**AICAR**	AMPK	Down-regulating Warburg and up-regulating OXPHOS	(170)
**SU212**	AMPK	Down-regulating Warburg and up-regulating OXPHOS	(171)
**Biguanide MC001**	AMPK/mTOR	Down-regulating Warburg and OXPHOS	(172)
**2-Deoxy-D-Glucose**	HK	Suppressing the Warburg effect	(173)

In addition, based on understanding the effect of ECM remodeling on TNBC invasive behavior, targeting ECM is becoming an emerging fulcrum for TNBC treatment protocols. For example, the TME-tunable BAGM therapeutic nanoplatform activates a cyclic cascade that degrades the dense ECM and converts matrix collagen into a loose state to exert antitumor effects (178). LOX inhibitor chemically linked lipid-based nanoparticles loaded with chemotherapeutic epirubicin have been demonstrated to prolong survival in patients with TNBC (179). This is probably because LOX inhibitors ablate the dense ECM and enhance chemotherapy drug permeability (62). Moreover, it has been demonstrated that inhibiting tumor development by targeting ECM to impact tumor cell metabolism is achievable (69, 180); the exact application of this technique to TNBC requires additional investigation.

The Warburg phenotype also functions as a strategy for eliminating transformed cells. RasV12-, Src-, or Erbb2-transformed cells are in a relatively rigid microenvironment formed by compacted epithelial cells, which are frequently apically extruded from epithelial tissues in search of more survival space, after which the epithelial defense against cancer (EDAC) induces epithelial protein lost in neoplasm (EPLIN)-mediated metabolic transformation (OXPHOS to glycolysis) to remove dissociative transformed cells (41, 181). This mechanism must be tested further to determine if it can be used to prevent the development of TNBC.

Worthy of note is that glycolysis suppression possibly triggers the up-regulation of compensatory metabolic pathways, such as OXPHOS (21), and drugs that simultaneously target both glycolysis and OXPHOS pathways, marizomib, and gracillin, have been successful in the mouse model (166, 182). It is necessary to clarify that while the inhibitory effect of targeted aerobic glycolysis on TNBC is undeniable, metabolic plasticity poses a new and pressing challenge for this therapeutic option (183). Perhaps targeting the metabolic switch is the key to tackling metabolic plasticity in the future (184).

## 5 Conclusion and perspective

Hypoxia is the source of the Warburg phenotype, whereas mutations and epigenetic modifications are the primary causes of its constitutive expression. The Warburg effect regulates TNBC invasion, including proliferation, metastasis, recurrence, treatment resistance, and immune evasion. Consequently, targeting the Warburg effect seems to be a promising method for treating refractory TNBC. Numerous *in vitro* investigations have also demonstrated the efficacy of this treatment protocol. Nevertheless, because of metabolic plasticity, the outcome of using a single glycolytic inhibitor is frequently disappointing (21, 183). Consequently, future research may focus less on a single glycolytic inhibitor delivery strategy and more on the following topics: **a.** A stratified approach to the management of TNBC patients based on metabolomics. **b.** Continuous innovation for auxiliary diagnostic technology of glycolytic-dependent TNBC. The simultaneous characterization of the Warburg effect using PET, MRI, and hyperpolarized ^13^C-MRSI is a notable advancement in this field (185). **c.** Targeting hypoxia. A hypoxic environment induces HIF to catalyze the Warburg phenotype, hence inhibiting HIF holds the promise of blocking glycolytic dysregulation at the root. **d.** Targeting ECM. The ECM regulates the expression of the Warburg effect, and the compacted ECM impacts chemotherapeutic drugs’ penetration; thus, the ECM-targeting protocol is significant for TNBC. **e.** Targeting multiple metabolic pathways. Since a single application of glycolysis inhibitors activates compensatory energy metabolic pathways, simultaneous inhibition of multiple metabolic pathways hopefully promotes apoptosis in cancer cells; Marizomib and gracillin, compounds that target both glycolysis and OXPHOS, have been successfully tested in mice. **f.** To develop new strategies to overcome the chemoresistance of TNBC by inhibiting the Warburg effect. **g.** Combined therapies. Chemoresistance of TNBC is the greatest barrier to chemotherapy treatments, and the Warburg effect certainly exacerbates this resistance. Theoretically, combining chemotherapeutic medicines and glycolytic inhibitors could increase the effectiveness. In addition, compacted ECM impedes drug delivery; hence ECM remodeling inhibitors paired with tumor cytotoxic medicines have the potential to boost TNBC patient survival.

## Author contributions

ML provided direction and guidance throughout the preparation of this manuscript. QS provided guidance throughout the revision of this manuscript. SJL collected and prepared the related literature and drafted the manuscript. YXL designed the imagines and tables. MY corrected the language ML and QS reviewed and made significant revisions to the manuscript. All authors contributed to the article and approved the submitted version article and approved the submitted version.
